# Omadacycline Potentiates Clarithromycin Activity Against *Mycobacterium abscessus*

**DOI:** 10.3389/fphar.2021.790767

**Published:** 2021-12-08

**Authors:** Bui Thi Bich Hanh, Nguyen Thanh Quang, Yujin Park, Bo Eun Heo, Seunghyeon Jeon, June-Woo Park, Jichan Jang

**Affiliations:** ^1^ Division of Applied Life Science (BK21 Four Program), Research Institute of Life Science, Gyeongsang National University, Jinju, South Korea; ^2^ Division of Life Science, Department of Bio & Medical Big Data (BK21 Four Program), Research Institute of Life Science, Gyeongsang National University, Jinju, South Korea; ^3^ Division of Life Science, Gyeongsang National University, Jinju, South Korea; ^4^ Department of Environmental Toxicology and Chemistry, Korea Institute of Toxicology, Korea & Human and Environmental Toxicology Program, Korea University of Science and Technology (UST), Daejeon, South Korea

**Keywords:** *Mycobacterium abscessus*, combination therapy, synergisctic effects, drug—drug interaction, novel combination therapy

## Abstract

*Mycobacterium abscessus* is a difficult respiratory pathogen to treat, when compared to other nontuberculus mycobacteria (NTM), due to its drug resistance. In this study, we aimed to find a new clarithromycin partner that potentiated strong, positive, synergy against *M. abscessus* among current anti-*M. abscessus* drugs, including omadacycline, amikacin, rifabutin, bedaquiline, and cefoxitine. First, we determined the minimum inhibitory concentrations required of all the drugs tested for *M. abscessus* subsp. *abscessus* CIP104536^T^ treatment using a resazurin microplate assay. Next, the best synergistic partner for clarithromycin against *M. abscessus* was determined using an *in vitro* checkerboard combination assay. Among the drug combinations evaluated, omadacycline showed the best synergistic effect with clarithromycin, with a fractional inhibitory concentration index of 0.4. This positive effect was also observed against *M. abscessus* clinical isolates and anti-*M. abscessus* drug resistant strains. Lastly, this combination was further validated using a *M. abscessus* infected zebrafish model. In this model, the clarithromycin-omadacyline regimen was found to inhibit the dissemination of *M. abscessus,* and it significantly extended the lifespan of the *M. abscessus* infected zebrafish. In summation, the synergy between two anti-*M. abscessus* compounds, clarithromycin and omadacycline, provides an attractive foundation for a new *M. abscessus* treatment regimen.

## Introduction

*Mycobacterium abscessus* (hereafter referred as *Mab*) is a deadly, drug-resistant, nontuberculous mycobacteria (NTM), that has been increasing in prevalence worldwide (Pan et al., 2017). In the United States and Korea, the *Mycobacterium avium* complex is the most common pathogen group causing NTM lung diseases, followed by *Mab* (Koh et al., 2011; Adjemian et al., 2018). *Mab* causes respiratory infections in patients whom are immunodeficient, have cystic fibrosis, are human immunodeficiency virus positive, have chronic obstructive pulmonary disease, or have bronchiectasis. It should be noted that pulmonary infection can also rarely occur in immunocompetent individuals with normal lung airways (Ryan and Byrd, 2018; Alramadhan et al., 2021). There are several recommended treatments for *Mab* infections. In 2017, the British Thoracic Society (BTS) guidelines recommended a revised antibiotic therapy comprised of intravenous amikacin (AMK), tigecycline (TGC), and imipenem (IMP) administered with a macrolide, for the initial treatment phase. For the continuation phase, nebulized AMK and a macrolide were used in combination with one to three of the following oral antibiotics: linezolid, clofazimine (CFZ), minocycline cotrimoxazole, and moxifloxacin (Haworth et al., 2017). Recent American Thoracic Society (ATS) guidelines also recommended a revised antibiotic therapy for *Mab* treatment. The initial treatment phase comprises parental drugs (AMK, IMP or cefoxitin, TGC) and oral drugs (Azithromycin; AZT or CLA, CFZ, Linezolid). For the continuation phase, oral drugs such as AZT or CLA, CFZ, linezolid, and inhaled AMK were combined. The number of drugs for treatment can be determined by *Mab* macrolide susceptibility testing (Kurz et al., 2020). The most striking difference is that the 2017 BTS still includes some fluoroquinolones while the 2020 ATS recommendations have removed fluoroquinolones from the list. However, *Mab* is resistant to many antibiotics including many in the currently implemented regimen, thus making it difficult to cure, and sometimes impossible to treat. Therefore, novel alternative regimens are urgently required.

Macrolides, such as clarithromycin (CLA) and azithromycin, are the main components of *Mab* treatment. Macrolides inhibit the growth of *Mab* by binding to the 23S ribosomal RNA to block bacterial protein synthesis (Stout and Floto, 2012). Thus, macrolides inhibit protein synthesis in bacteria at an early stage of translation (Zhang et al., 2017). Although macrolides are the cornerstone agents of the multidrug therapy approach for treating *Mab*, the effectiveness of macrolides are not satisfactory due to the prevalence of macrolide-resistant strains. For example, *in vitro*, 3 days after the exposure of *Mab* to CLA, inducible resistance is generated. After 14 days, CLA shows lower inhibitory activity against *Mab* (Nie et al., 2014). *Mab.* subsp. *abscessus* and *Mab*. subsp. *bolletii* are capable of inducing resistance by up-regulation of the functional erythromycin ribosomal methylase gene, *erm*(41). However, *Mab.* subsp. *massiliense* usually possesses non-functional *erm*(41) gene copies that have a 274-bp deletion, and, hence, are susceptible to macrolides (Kim et al., 2010). Because of this, there is an urgent need for a new macrolide-based combination regimen. In particular, it is important to identify other drugs that are synergistic with CLA against *Mab*. To identify such a synergy, we evaluated the effect of a CLA-omadacycline (OMD) combination against the *Mab* complex *in vitro* and using a zebrafish model.

## Materials and Methods

### Bacterial Strains/Culture Conditions/Chemicals

*Mab* subsp. *abscessus* CIP 104536^T^ S and R morphotypes were kindly provided by Dr. Laurent Kremer (CNRS, IRIM, Universite’ de Montpellier, Montpellier, France). *Mab* subsp. *bolletii* CIP108541^T^ and *Mab* subsp. *massiliense* CIP108297^T^ were purchased from the Collection de l’Institut Pasteur (CIP, Paris, France). Clinical isolates were obtained from the Korea *Mycobacterium* Resource Center (KMRC, Osong, Korea). AMK and CFX resistant strains used in this study were derived from a previous study (Kim et al., 2017). *Mab* strains were grown at 37°C in a Middlebrook 7H9 culture medium (Difco), supplemented with 10% albumin-dextrose-catalase (ADC, Difco) and 0.05% Tween-80 (Sigma). For the CFU determination, bacteria was plated in a Middlebrook 7H10 solid culture medium containing 0.5% glycerol and 10% OADC (Difco). In order to evaluate the MIC and drug-drug interaction, the bacteria was tested in a cation-adjusted Mueller–Hinton (CAMH) medium (Sigma, St. Louis, MO, United States) supplemented with 20 mg/L calcium chloride (Sigma, St. Louis, MO, United States) and 10 mg/L magnesium chloride (Sigma, St. Louis, MO, United States). To induce the zebrafish infection, a recombinant *Mab* CIP 104536^T^ R morphotype that was carrying a pMV262-mWasabi, that was prepared previously, was used (Kim et al., 2019). All cultures were grown at 37°C while shaking at 180 rpm. CLA, RFB, AMK, and CFX were purchased from Sigma-Aldrich (St. Louis, MO, United States). OMD and BDQ were purchased from Adooq Bioscience (Irvine, CA, United States).

Determination of compound interactions using a REMA checkerboard assay and evaluation of compound interactions using CFU determination

For each drug’s MIC determination, a REMA was performed, as described previously (Hanh et al., 2020a). Furthermore, checkerboard assay using resazurin was performed in a similar manner to that described for *Mab* by Cheng *et al.*,with minor modifications (Cheng et al., 2019). The checkerboard method was used to evaluate the antibacterial ability of the two antibacterial drugs. 1 µL of the two-fold serial dilutions of each test compound (starting from 8 × the MIC_50_) was prepared in a well of a 96-well flat, clear bottom, white microplate (98 µL per well) (Corning, Baltimore, MD, United States). Bacterial stocks of *Mab* subsp. *abscessus* CIP 104536^T^ from the exponential-phase cultures were eluted to an optical density measuring 600 nm (OD_600_) of 0.0025 and added to the plates to obtain a total volume of 100 µL. Each plate was then incubated for 5 days at 30°C, before the addition of resazurin [0.025% (wt/vol) to 1/10 of well volume] as described previously (Cheng et al., 2019). After overnight incubation, fluorescence was measured using a spectraMax® M3 Multi-Mode Microplate Reader (Molecular Devices, Sunnyvale, CA, USA) with excitation at 560 nm and emission at 590 nm.

To evaluate compound interactions, fractional inhibitory concentrations (FICs) were calculated using the following formula: FIC (X + Y) = [MIC of compound X in combination with Y]/[MIC of X alone]. The fractional inhibitory index (ΣFIC) is the sum of the FIC of compound X and the FIC of compound Y. Synergy was defined by ΣFIC values of ≤0.5, antagonism by ΣFIC values> 4.0, and values in between correspond to additivity (Odds, 2003). The isobologram curves showing the result of the interaction of the two antibacterial agents from the MICs for the antibacterial agents when used alone, or in combination, were constructed using GraphPad Prism software (version 6.05; San Diego, CA, United States). To detect the bacterial viability, bacteria was first incubated in the presence of combinations of the compounds at their respective MICs before they were then plated on solid Middlebrook 7H10 mediums (Difco). CFU counts were determined after 3 days of incubation at 37°C.

### Ethics

All ZF experiments were approved by the Animal Research Ethics Committee of Gyeongsang National University (Project identification code: GNU-190325-E0014, Approval date: Mar 25, 2019).

### Zebrafish Infection and Drug Treatment

*Mab* CIP 104536^T^ R morphotype, harboring mWasabi, was selected under the pressure of kanamycin 50 mg/L. The infection stock was prepared as described previously (Hanh et al., 2020b). Infection stock was then diluted with PBST (Phosphate-Buffered Saline with 0.05% Tween 80) and re-suspended in Phenol Red 0.085%. The zebrafish larvae at 30–48 h post-fertilization were dechorionated and anesthetized with 270 mg/L tricaine at room temperature. Around 3 nL of *Mab*R-mWasabi (400 CFU) was injected via the caudal veins using a Tritech Research Digital microINJECTOR (Tritech research, model MINJ-D). The infected larvae were transferred into 96-well plates (2 fish per well containing 200 µL water) and exposed to various drug combinations. CLA (3.1 µM) was combined with other anti-*Mab* agents (OMD 6.3 µM, BDQ 3.1 µM, AMK 12.5 µM, RFB 6.3 µM, and CFX 6.3 µM). The fish water and compounds were renewed once daily. The ZF larvae treated with DMSO as a vehicle were used as a negative control.

### Drug Efficacy Assessment in MabR-mWasabi Infected ZF

ZF *in vivo* drug efficacy was assessed as described previously (Hanh et al., 2020a). Briefly, the *in vivo* anti-*Mab* effect of each drug combination was determined through GFP dissemination, counts of CFU, and the survival curve. GFP quantification was accessed by capturing the *Mab*R-mWasabi evolution inside the infected larvae at 5 days post-infection using an ImageXpress Pico Automated Cell Imaging System (Molecular Devices, Sunnyvale, CA, United States). For the quantification of bacterial load, a group of 20 infected embryos (5 dpi) was collected and individually homogenized in 2% Triton X-100–PBST using a handheld homogenizer (D1000; Benchmark Scientific, Sayreville, NJ, United States). Serial 10-fold dilutions of the suspension were plated out on Middlebrook 7H10 solid culture mediums containing 50 μg/ml kanamycin and BBL™ MGIT™ Mycobacteria Growth Indicator PANTA (polmyxin B, amphotericin B, nalidixic acid, trimethoprim, and azlocillin; Becton Dickinson, Franklin Lakes, NJ, United States), and then incubated for 3–5 days at 37°C to enumerate the CFU. The number of dead embryos (no heartbeat) was recorded daily, for 13 days, to determine the survival curve. The CFU quantification and survival curve were plotted by Prism using the method from Kaplan and Meier and the log-rank (Mantel–Cox) test, respectively, to compare the difference between untreated control and treated embryos.

## Results

### Checkerboard Assay for Compound Interactions

**FIGURE 1:**
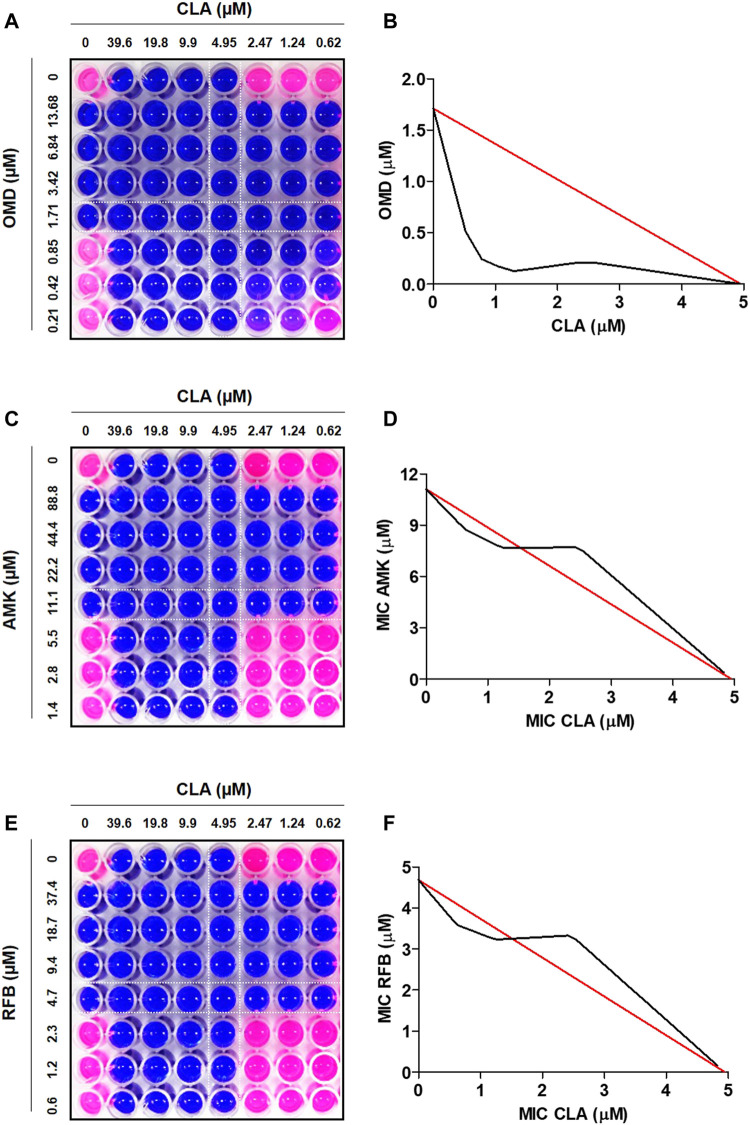
Drug-drug interaction using checkerboard assay. Drug interaction was evaluated in MIC_50_, one-half the MIC_50_, one-quarter the MIC_50_, *one half* of *one quarter the* MIC_50_ of CLA (horizontal) in combination with MIC_50_, one-half the MIC, one-quarter the MIC_50_, *one half* of *one quarter the* MIC_50_ of OMD **(A)**, AMK **(C)**, and RFB **(E)**. Isobolograms of the resazurin checkerboard synergy testing method showing synergy of CLA with OMD **(B)**. The additive effect observed when CLA interact with AMK **(D)** and RFB **(F)**. The white line indicates MIC_50_ value of each compound.

To find the best combination with CLA, various drugs were included in this experiment. First, the MIC_50_ value of each individual compound was determined by Resazurin Microtiter Assay (REMA). MIC_50_ value was defined as the minimum inhibitory concentration (MIC) required to inhibit 50% growth of the organism. The MIC values of all tested compounds are presented in Table 1. CLA showed favorable activity against *Mab* (MIC_50_ = 5 µM). Second, drug-drug interactions were evaluated to find the best combination with CLA, using mid-log phase cells of *Mab* utilizing a checkerboard assay. CLA concentrations ranging from 0 to 39.6 µM (8 points) were prepared in 96 well plates through 2-fold serial dilution, and the MIC_50_ values of CLA were placed at the middle of the concentration range. This gradient CLA concentration was used to test interactions with five different anti-*Mab* drugs such as OMD, AMK, rifabutin (RFB), BDQ, and CFX in various drug concentrations, based on 2-fold serial dilution. The interactions were interpreted using a fractional inhibitory concentration (FIC) index for each combination (Table 1). The experiment was repeated three times and the combination effect was consistent across the replicated experiments. As shown in Figure 1, the concentrations of CLA and OMD required for this synergistic effect were much lower than their MIC alone. For example, one-half the MIC_50_ of CLA (pink) added to one-half the MIC_50_ of OMD (pink) did not result in a resazurin color change in the dye from blue-purple to pink, which indicates the inhibition of *Mab* growth (Figures 1A,B). Furthermore, one-quarter the MIC_50_ of CLA added to one-quarter the MIC_50_ of OMD also prevented resazurin turnover. Synergy has traditionally been defined with a FIC index of 0.5 or less (Braga et al., 2005). Thus, the CLA plus OMD combination effect is synergistic against *Mab*. Interestingly, the CLA-BDQ combination also showed a synergistic effect (FIC = 0.5) against *Mab,* although the FIC index was higher than the CLA-OMD combination (Table 1; Supplementary Figures S1A, S1B). The CLA with AMK, RFB, and CFX combinations showed no synergistic antimicrobial effects, with a FIC value of over 0.5. CLA showed an additive effect with AMK (Figures 1C,D), RFB (Figures 1E,F), and CFX (Supplementary Figures 1C, 1D) against *Mab,* with FIC index values of 0.7–1.4. This means each compound did not interact in a direct way without affecting the other (Table 1). No antagonistic interactions were found between CLA and the compounds tested.

**TABLE 1:** MICs of selected anti-*M*. abscessus drugs against *M. abscessus* subsp. *abscessus* CIP 104536^T^ and corresponding interaction profiles with clarithromycin (CLA) evaluatedby REMA checkerboard.

Drungs	MIC_50_ (uM) by REMA	Interaction profile with CLA	
		∑FIC	Outcome
Clarithromycin (CLA)	5.0	—	—
Omadacycline (ODC)	1.7	0.4	Synergistic
Amikacin (AMK)	11.1	1.4	Additive
Rifabutin (RFB)	4.7	1.4	Additive
Bedaquiline (BDQ)	0.6	0.5	Synergistic
Cefoxitin (CFX)	0.2	0.7	Additive

### Colony Forming Unit Determination for Drug-Drug Interaction

**FIGURE 2:**
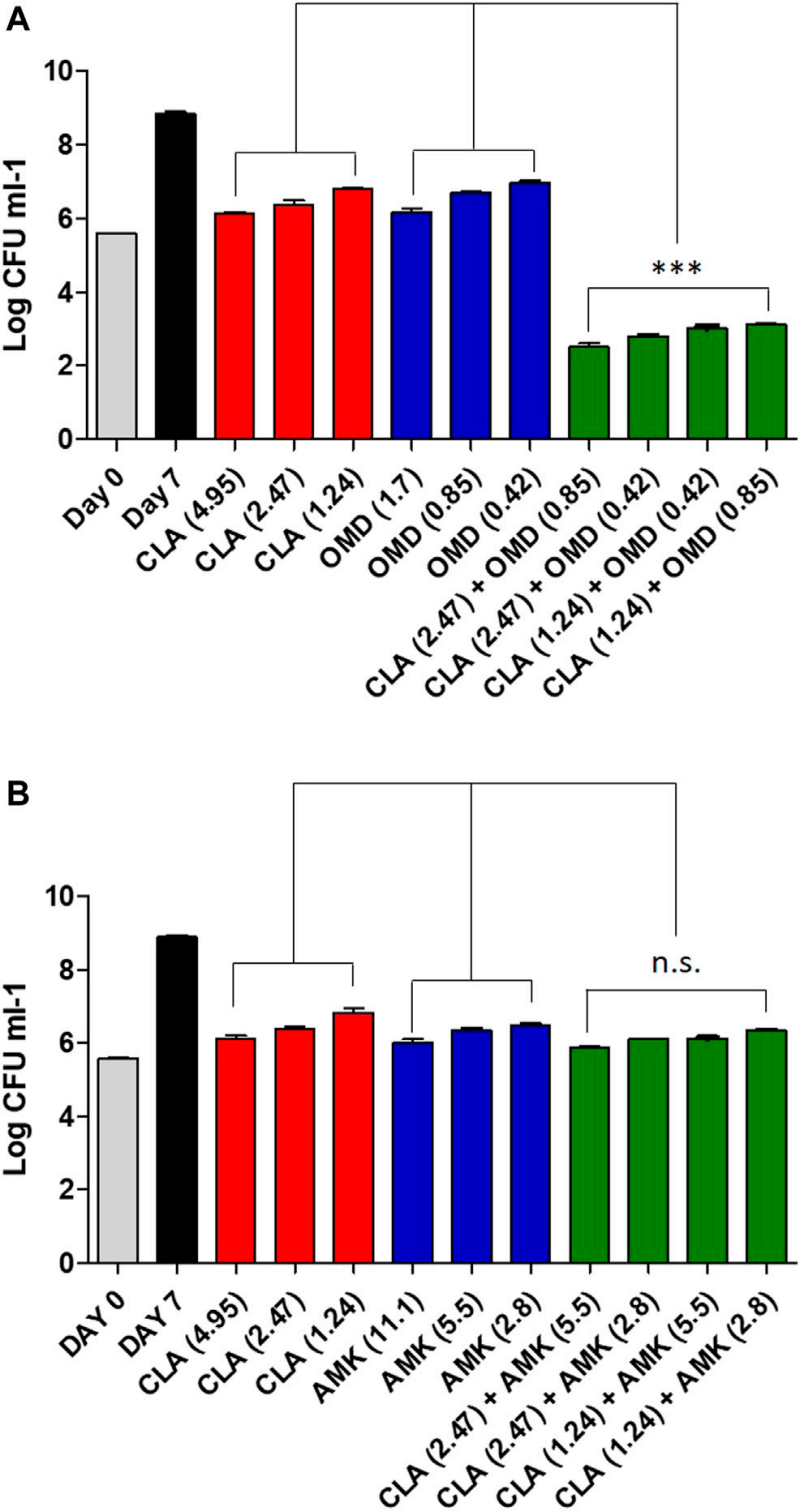
Estimation of bactericidal effect by CFU counts. *Mab* was grown in the presence of different concentrations of CLA alone or in combination with decreasing concentrations of OMD **(A)** and AMK **(B)**. Following 7 days of culture, *Mab* were plated to 7H10 agar plate to determine live bacteria. The DMSO treated bacteria were also plated on day 0 and on day 7. One-way ANOVA with Tukey’s multiple comparison test was used to compare the means across multiple groups (**p < 0.01; ***p < 0.001).

To confirm the synergistic effect against *Mab*, a traditional CFU (colony forming unit) determination assay was conducted. As shown in Figure 2A, the obtained results were consistent with the results from the checkerboard method. The CFU determination assay confirmed that combinations of CLA with OMD showed a clear synergistic effect leading to a significant reduction in bacterial numbers on the agar plates. The results show that the combination of 2.47 μM of CLA (one-half the MIC) and 0.85 µM OMD (one-half the MIC) had clear growth inhibitory activity (6.4 log_10_ cfu/mL reduction), compared to the activity of the untreated DMSO control on day 7. In addition, this combination also showed at least a 3 log_10_ cfu/mL reduction compared to the single CLA and OMD samples respectively. Based on the definition, bactericidal activity was defined as a reduction of at least ≥3 log_10_ of the total count of CFU/mL in the original inoculum. Therefore, the CLA plus OMD combination is shown to be bactericidal against *Mab* (Kragh et al., 2021). Again, the CLA-BDQ combination also showed significant bacterial reduction, as similar with the checkerboard assay. However, the CLA-BDQ combination showed less than a 3 log_10_ cfu/mL reduction compared to the single BDQ. Thus, this combination was considered to be bacteriostatic against *Mab* (Supplementary Figure S2A). Conversely, CLA in combination with AMK, RFB, and CFX acted additively, with the combinations giving similar inhibition of bacterial viability to the single agents (Figure 2A; Supplementary Figures S2B, S2C). Furthermore, we tested CLA-OMD effectiveness against *Mab* subspecies and clinical isolates that have different morphotypes, including AMK and CFX laboratory induced resistant strains that were generated in a previous study (Kim et al., 2017). Table 2 shows the MIC of CLA and OMD alone, and the FIC values in combination, against 3 *Mab* subspecies, 7 clinical isolates (6 *Mab* subsp. *abscessus* and 1 *Mab* subsp. *massiliense*), and AMK and CFX resistant strains. Synergism was found in 100% of the strains tested. Three different *Mab* R morphotypes (*Mab* subsp. *abscessus* CIP104536, KMRC 00136-61040, and KMRC 00200-61202) also showed a synergistic effect (FIC index less than 0.5).

**TABLE 2:** MICs and interaction profiles of clarithromycin (CLA) and omadacycline (OMD) against *M. abscessus* strains.

*Mab strains*	Colony morphotype	MIC (uM) by REMA		Interaction profile with CLA	
		CLA	OMD	∑FIC	Outcome
subsp. *abscessus* CIP104536	R	5.01	1.71	0.44	synergism
subsp. *massilience* CIP108297	S	3.87	1.70	0.39	synergism
subsp. *bolletii* CIP108541	S	5.50	1.68	0.46	synergism
subsp. *abscessus* KMRC 00136-61038	S	4.95	1.70	0.45	synergism
subsp. *abscessus* KMRC 00136-61039	S	4.25	1.75	0.43	synergism
subsp. *abscessus* KMRC 00136-61040	R	5.60	1.68	0.49	synergism
subsp. *abscessus* KMRC 00136-61041	S	4.73	1.74	0.44	synergism
subsp. *abscessus* KMRC 00200-61199	S	5.20	1.65	0.47	synergism
subsp. *abscessus* KMRC 00200-61200	S	4.95	1.73	0.47	synergism
subsp. *massiliense* KMRC 00200-61202	R	5.40	1.70	0.49	synergism
subsp. *abscessus* CIP104536 (CFX-R)	S	4.92	1.68	0.43	synergism
subsp. *abscessus* CIP104536 (AMK-R)	S	4.90	1.71	0.44	synergism

### Activity of Clarithromycin and Omadacycline Combinations in Mab Infection in Zebrafish

**FIGURE 3:**
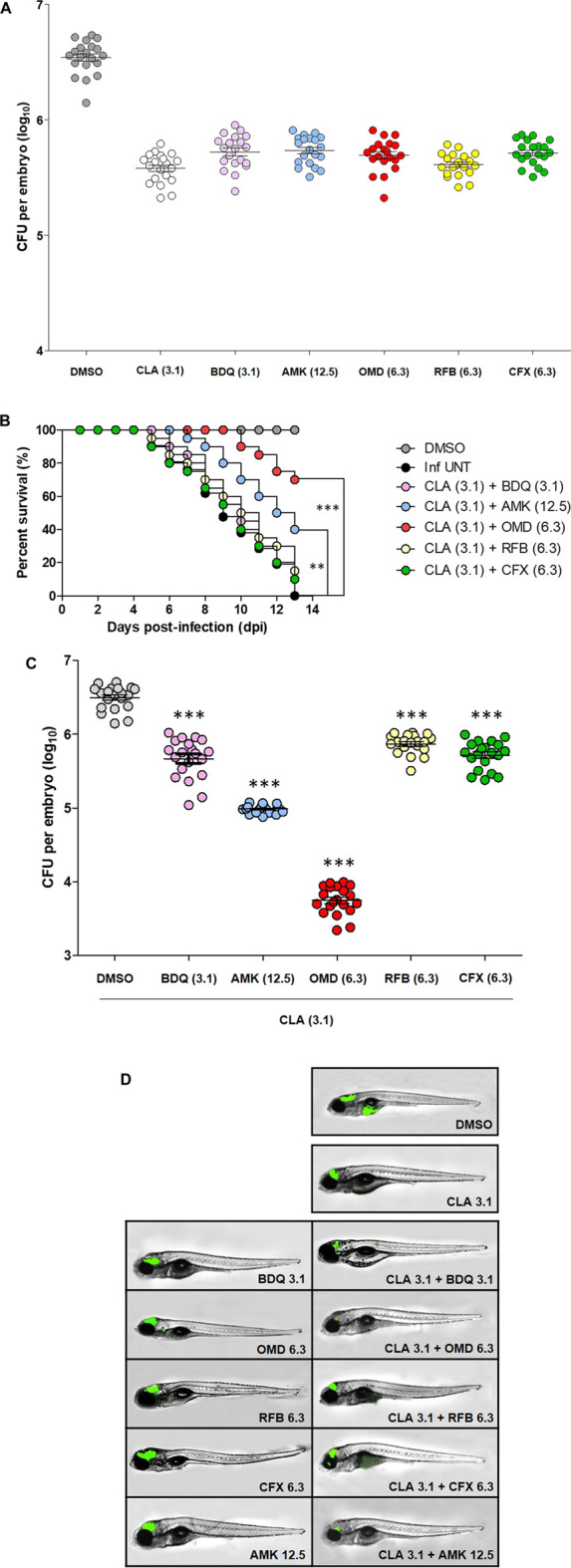
ZF *in vivo* efficacy of CLA-OMD. The drug concentrations that show 1 log_10_ CFU reduction were determined using different concentrations of CLA, BDQ, AMK, OMD, RFB, and CFX in *Mab* infected ZF model **(A)**. To determine *in vivo* efficacy, survival curve was plotted from *Mab*R-mWasabi infected ZF for 13 days (*n* = 20, representative of three independent experiments) **(B)**. Each different combination treatment was carried out. CLA (3.1 μM) was combined with BDQ (3.1 μM), AMK (12.5 μM), OMD (6.3 μM), RFB (6.3 μM), and CFX (6.3 μM) respectively. Survival curves were constructed using the log-rank (Mantel-Cox) test (**p < 0.01; ***p < 0.001). Inf UNT: Infected but not treated control. Therapeutic outcome using drug combinations was validated by traditional agar plate quantification method **(C)**. Data was expressed as the mean log_10_ CFU per embryo (*n* = 10 of each condition) from three independent experiments. Drug combination effect was also observed using fluorescence under microscope. Each drug combinations were treated to the ZF infected with *Mab*R-mWasabi and reduction of mWasabi signal in ZF was monitored under the fluorescent microscope **(D)**.

Furthermore, we verified the CLA-OMD combination effectiveness in *Mab*-infected ZF. To do this, the dilution of each drug evaluated was set as the concentration that resulted in a reduction of 1 log_10_ CFU in infected ZF survival, compared to the untreated control, by serial drug dilution at 5 days post-infection (dpi) (data not shown). From this drug serial dilution, CLA (3.1 µM), BDQ (3.1 µM), AMK (12.5 µM), OMD (6.3 µM), RFB (6.3 µM), and CFX (6.3 µM) were determined as the drug concentrations that yielded approximately a 1 log_10_ CFU reduction on an agar plate, when compared to the untreated control (Figure 3A). For the next step, an *in vivo* combination drug efficacy test was performed with these selected concentrations of each drug. CLA was used as the anchor drug and was separately paired with BDQ, AMK, OMD, RFB, and CFX. As shown in the survival curve (Figure 3B), double therapy with CLA (3.1 µM) and OMD (6.3 µM) yielded a significantly lower mortality rate than the other combinations. The CLA plus OMD combination led to a 30.5% mortality rate at 13 days after treatment. In contrast, the conventional pairing of CLA (3.1 µM) plus AMK (12.5 µM), or CFX (6.3 µM), showed a higher mortality rate (60.5 and 90% of *Mab*-infected ZF at 13 dpi, respectively). The combination of CLA (3.1 µM) plus BDQ (3.1 µM) was also not effective. It showed a 10% survival rate for infected ZF at 13 dpi. Furthermore, the combination between CLA and RFB also showed almost no synergistic effect. The *Mab* infected and untreated ZF group 100% died and the non-infected group 100% survived after 13 days.

The bacterial burden in the ZF was measured by conventional CFU counts and fluorescence microscopy after different combinations of treatments. To determine whether each combination effectively reduced the bacterial burden in the ZF, bacterial survival was compared with the CLA (3.1 µM) and OMD (6.3 µM) treatments and the other CLA combinations (BDQ, AMK, RFB, and CFX), including the non-treated DMSO control and non-infected ZF. To do this, each infected and treated ZF was crushed and sampled, and the number of bacteria was enumerated on a 7H10 agar plate. Figure 3C shows that *Mab* replicated inside the hosts, and the CFU showed significant differences between the groups. The lowest bacterial CFU per ZF was observed in the presence of CLA (3.1 µM) and OMD (6.3 µM) as expected (Figure 3C). This combination showed around a 2.8 log_10_ reduction compared with the non-treated DMSO control group 5 days after injection. This result was consistent with the observed survival rate in infected ZF (Figure 3B). To confirm the colonization of *Mab* inside the ZF bodies under treatment with different combinations, we also used a mWasabi green fluorescent protein (GFP) labeled *Mab* strain. The GFP levels were observed using an ImageXpress® Pico Automated Cell Imaging System on anaesthetized ZF. It allowed us to measure the progression of GFP labelled bacterial colonization following combination treatment. ZF treated with CLA (3.1 µM) plus OMD (6.3 µM) were compared with those treated with other combinations and control ZFs. GFP labelled *Mab* dissemination was observed in the brain and yolk in the non-treated DMSO control (Figure 3D). However, ZFs treated with CLA (3.1 µM) and OMD (6.3 µM) showed almost no GFP fluorescence (Figure 3D). GFP signals in the brain area were still observed in other CLA combinations, although the GFP signal in the ZF yolks disappeared. These results were consistent with the survival curves and the CFU determination (Figures 3B,C). Therefore, these results indicate that the combination of CLA and OMD significantly inhibited *Mab* growth in the ZF bodies and, consequently, extended the lifespan of the infected ZF.

## Discussion

Although some antibiotics, such as AMK, CFX, and IMP, show effectiveness against *Mab*, only CLA shows persuasive evidence of clinical efficacy for the treatment of pulmonary disease caused by *Mab* (Nie et al., 2014). For this reason, CLA is currently the only effective antibiotic for oral administration. Therefore, it is recommended as the main agent for treatment of *Mab* infections (Guo et al., 2018). Current treatment of *Mab* infections consist of a CLA based regimen including AMK and either CFX or IPM. However, current treatment outcomes are extremely unsatisfactory. Based on the meta-analysis performed by Diel *et al.,* the clinical treatment success rate of *Mab* pulmonary disease is generally 41%. Thus, some *Mab* infected patients were also subjected to adjunctive surgery (Diel et al., 2017). A possible explanation for this low clinical treatment success rate may be due to a gene named *erm* (41) that is involved in CLA resistance against *Mab*. The macrolide-resistant ability of *Mab* (especially *Mab* subsp. *abscessus* and *Mab* subsp. *bolletii*) is induced by an adaptive resistance mechanism using the inducible ribosomal methylase *erm*(41) gene. Although, *Mab* subsp. *massiliense* isolates contain a truncated *erm*(41) gene that has shown improved clinical treatment outcomes (Bronson et al., 2021; Quang and Jang, 2021). Furthermore, CLA treatment induces the expression of transcriptional regulator WhiB7, which causes upregulation of *erm*(41) and *eis*2 (which provides AMK resistance) (Pryjma et al., 2018). For these reasons, there are some doubts for use of CLA as the main component in the current regimen against *Mab* treatment. According to a meta-analysis using literature published between 1990 and 2017, macrolide-containing regimens achieved sustained sputum culture conversion (SSCC) in 34% new *Mab* subsp. *abscessus* patients versus 54% *Mab* subsp. massiliense patients. In refractory disease, SSCC was achieved in only 20% of patients across all subspecies (Pasipanodya et al., 2017). Although these outcomes that were from currently recommended regimens look atrocious, there is no viable alternative because of no potent anti-*Mab* candidates that were approved its efficacy in humans through clinical trials. In this perspective, it is clear that there is an urgent need for discovering and developing novel, more innovative anti-*Mab* drugs (Quang and Jang, 2021). Therefore, there have been many attempts to find the best partner for CLA to improve treatment outcomes. For example, a CLA plus TGC combination was tested on *Mab* complex isolates, which showed synergistic effectiveness. Combined CLA with TGC was highly synergistic against *Mab* subsp. *abscessus*, *Mab* subsp. *massiliense*, and *Mab* subsp. *bolletii* isolates (Huang et al., 2013; Zhang et al., 2017). TGC has been spotlighted for *Mab* treatment, with moderate *in vitro* activity against most clinical isolates of *Mab* (MIC_90;_ 2–16 mg/L). It sometimes is used as a supplement to triple antibiotic therapy when current regimens are ineffective (Singh et al., 2014; Pryjma et al., 2018; Kaushik et al., 2019; Kwon et al., 2019). However, TGC treatment has resulted in severe adverse effects, such as nausea and vomiting (Chen et al., 2019; Quang and Jang, 2021). Thus, intravenous administration of TGC is not desirable for long-term treatment (Kaushik et al., 2019). Therefore, a new version of TGC, with similar or better efficacy and fewer adverse effects, preferably with oral bioavailability, is required to improve the treatment outcome for *Mab* infections (Kaushik et al., 2019).

OMD is an alternative desirable TGC analog. On October 2, 2018, OMD was approved by the US Food and Drug Administration (FDA) for the treatment of adults with community-acquired bacterial pneumonia and acute skin, and skin structure, infections (Markham and Keam, 2018). OMD has shown positive *in vitro* activity against *Mab* with promising results (MIC_90_; 2 mg/L) (Kaushik et al., 2019; Nicklas et al., 2021). Recently, *in vivo* efficacy of OMD evaluated at a dose equivalent to the 300 mg standard oral human dose showed a 1 to 3 log10 reduction in bactericidal activity against all tested *Mab* strains, compared to an untreated control group (Nicklas et al., 2021). Considering the steady-state area under the curve (AUC), and MICs obtained against *Mab*, the free drug AUC/MIC ratios for OMD, given intravenously, is expected to be approximately eight to ten times higher than TGC (Kaushik et al., 2019). Therefore, this improves the intravenously administered pharmacokinetic/pharmacodynamic parameters, and the activity data suggests that OMD could be more effective than TGC in clinical treatment (Kaushik et al., 2019). OMD also shows significantly less occurrences of nausea and fewer treatment-emergent adverse events than TGC. Recently, a clinical study reported on the use of OMD on four patients with culture-positive *Mab* disease (two patients had cutaneous disease, one had pulmonary disease, and another had osteomyelitis and bacteraemia). In this study, the patients were treated with an OMD regimen, including other antimicrobial agents, for a median duration of 166 days. OMD-containing regimens showed a clinical cure in three of the 4 patients. The side effects of OMD were relatively tolerable during long-term treatment (Pearson et al., 2020).

In this study, we also showed that the MICs of CLA and OMD against *Mab* were significantly reduced by the administration of a CLA-OMD combination. The impact of the CLA was assessed *in vitro* by determining the inhibitory activity of various drug combinations. CLA combined with OMD was highly active *in vitro*, leading to a 0.4 FIC value. Recently published two articles also discovered an *in vitro* synergistic effect of CLA-OMD against *Mab* similar to this study (Gumbo et al., 2020; Nicklas et al., 2021). In the Nicklas *et al.* study, OMA in combination with CLA exhibited synergy against a *Mab* clinical isolate with a FIC index of ≤0.5 (Nicklas et al., 2021). This result strongly supports our new findings for this study.

This new finding was further validated using ZF larvae infected by *Mab* microinjection into the caudal vein. ZF share a high degree of genetic similarity with humans, and approximately 70% of all human disease genes show functional homologs in ZF (Santoriello and Zon, 2012; Nie et al., 2020). Furthermore, ZF are relatively simple to work with, cost-effective, and have genetic tractability and optical transparency, which allows for very easy and valid research. Of course, there are some examples of drugs that are effective in humans but not in ZF, and vice versa. However, evidences that have been accumulated more than 20 years in drug screening using ZF indicates that drugs which are work in ZF are similarly active in mouse and human systems with similar pharmacokinetic (PK) properties (Patton et al., 2021). Therefore, the *Mab*/ZF embryo model has been widely used for infectious diseases pathogenesis, especially for the assessment of antibacterial (Bernut et al., 2014; Hanh et al., 2020a, 2020b; Sullivan et al., 2021). It should be noted that the ZF model has some limitations in comparison with mammalian models. For example, ZF has gills instead of lungs and a lack of adaptive immunity in early development. Therefore, early-embryo infection models are more suitable for studying acute *Mab* infections, rather than chronic diseases (Bernut et al., 2017). Utilizing an *in vivo* early-embryo infection model, we injected *Mab* through the caudal vein and initiated treatment with CLA alone, or in combination with the various anti-*Mab* agents. In this study, CLA alone reduced the CFU of *Mab* in ZF embryos, and the efficacy of the CLA was significantly improved by the addition of OMD (Figure 3). Survival curves show that the CLA and OMD combination was the most effective at increasing *Mab*-infected ZF survival rate (70% survival after 13 dpi; Figure 3B). The evaluation of the efficacy of the CLA based drug combinations *in vitro*, and in ZF, indicate that the synergistic combination of CLA and OMD should be evaluated in more complex organisms, such as in immunocompromized rodents.

## Data Availability

The original contributions presented in the study are included in the article/Supplementary Material, further inquiries can be directed to the corresponding author.
