# Supplementary material for: Omadacycline Potentiates Clarithromycin Activity Against Mycobacterium abscessus
Source: Front Pharmacol. 2021 Dec 8;12:790767. doi: 10.3389/fphar.2021.790767 (PMC8693020; doi:10.3389/fphar.2021.790767)
Supplement: Supplementary file 1 [file Presentation1.pptx]

## Slide 1
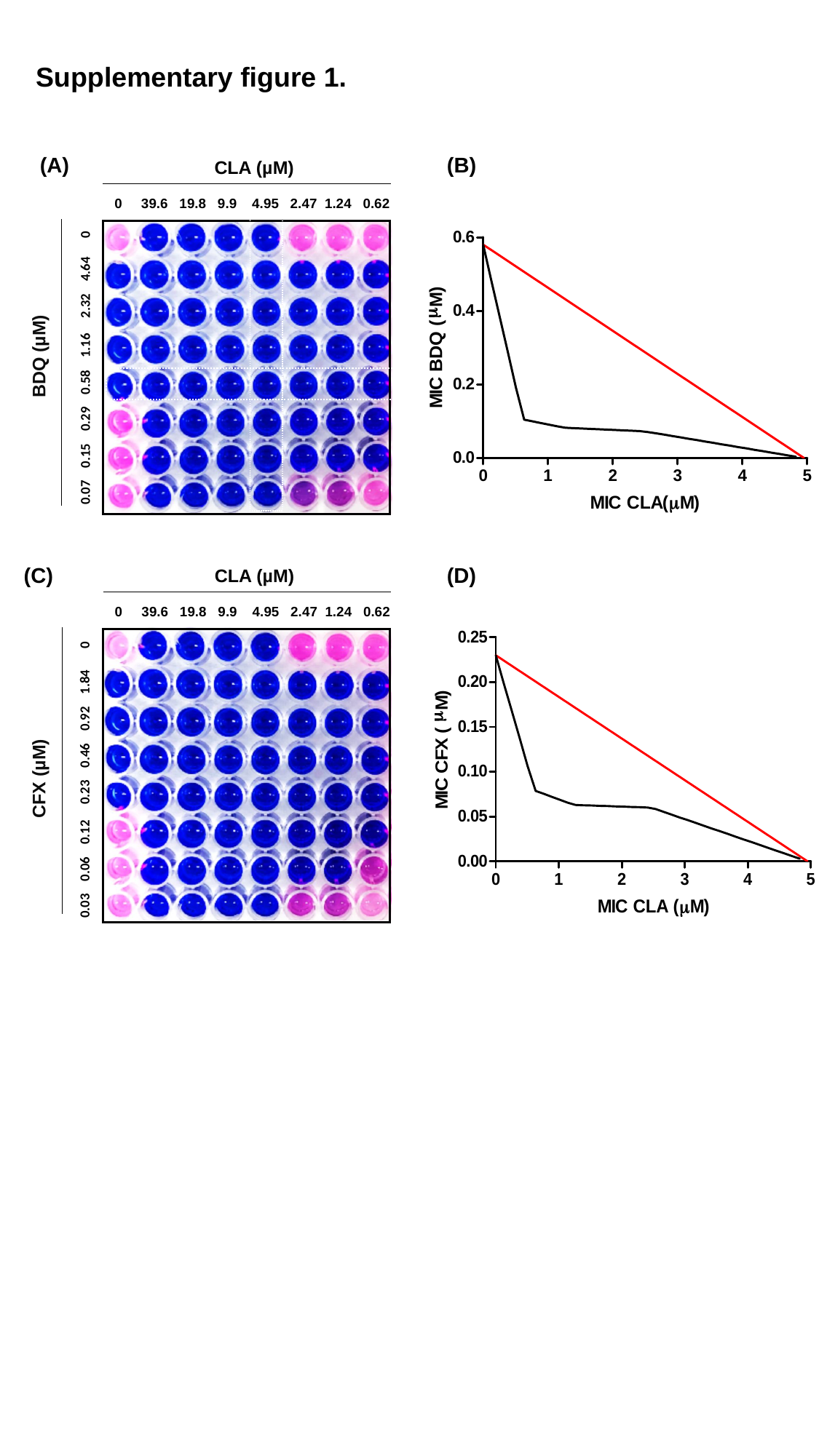

Supplementary figure 1.
(A)
(B)
CLA (µM)
Telacebec (Q203)
Telacebec (Q203)
0 39.6 19.8 9.9 4.95 2.47 1.24 0.62
 0.07 0.15 0.29 0.58 1.16 2.32 4.64 0
BDQ (µM)
(C)
(D)
CLA (µM)
Telacebec (Q203)
Telacebec (Q203)
0 39.6 19.8 9.9 4.95 2.47 1.24 0.62
0.03 0.06 0.12 0.23 0.46 0.92 1.84 0
CFX (µM)

## Slide 2
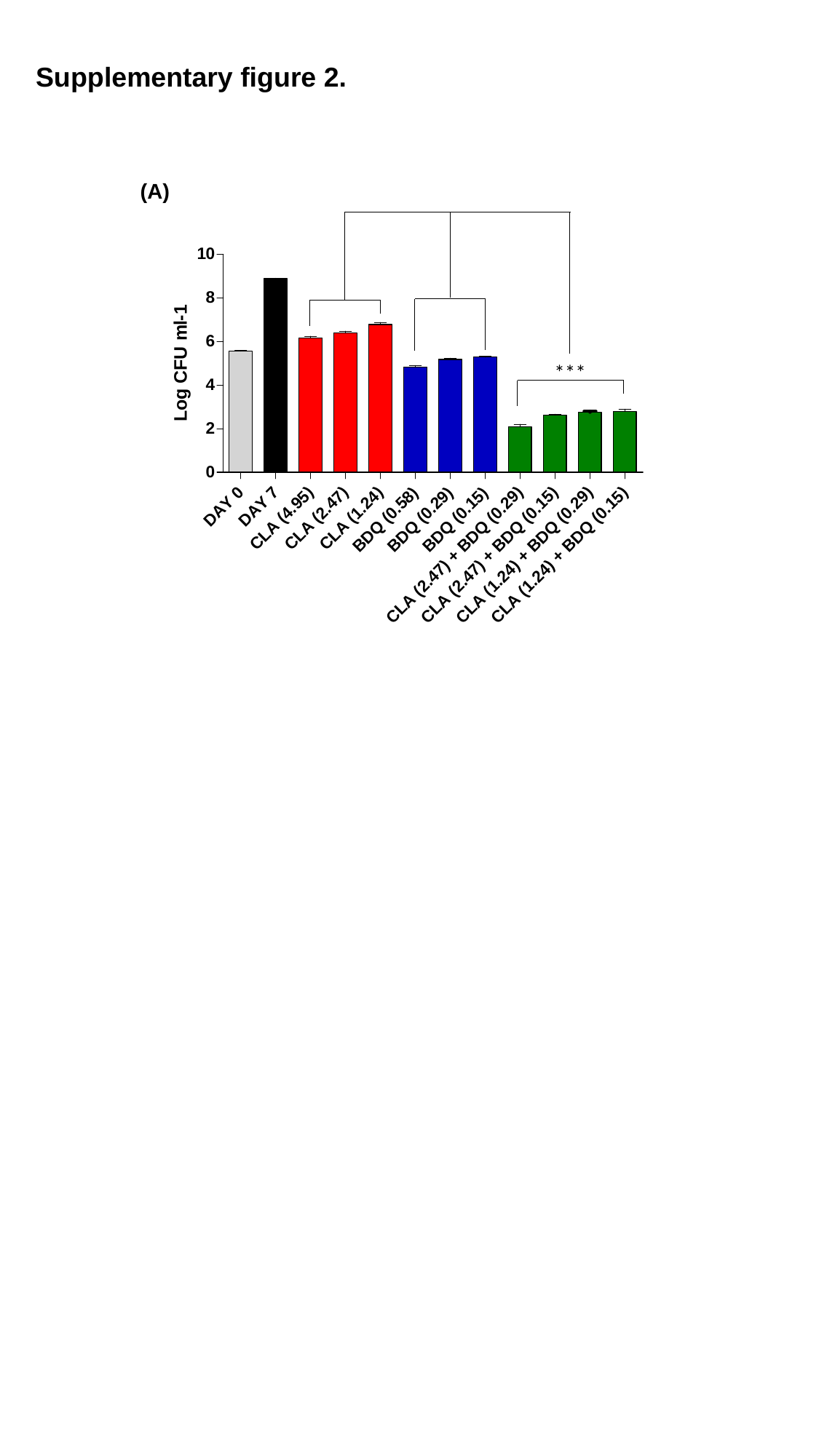

Supplementary figure 2.
(A)
***

## Slide 3
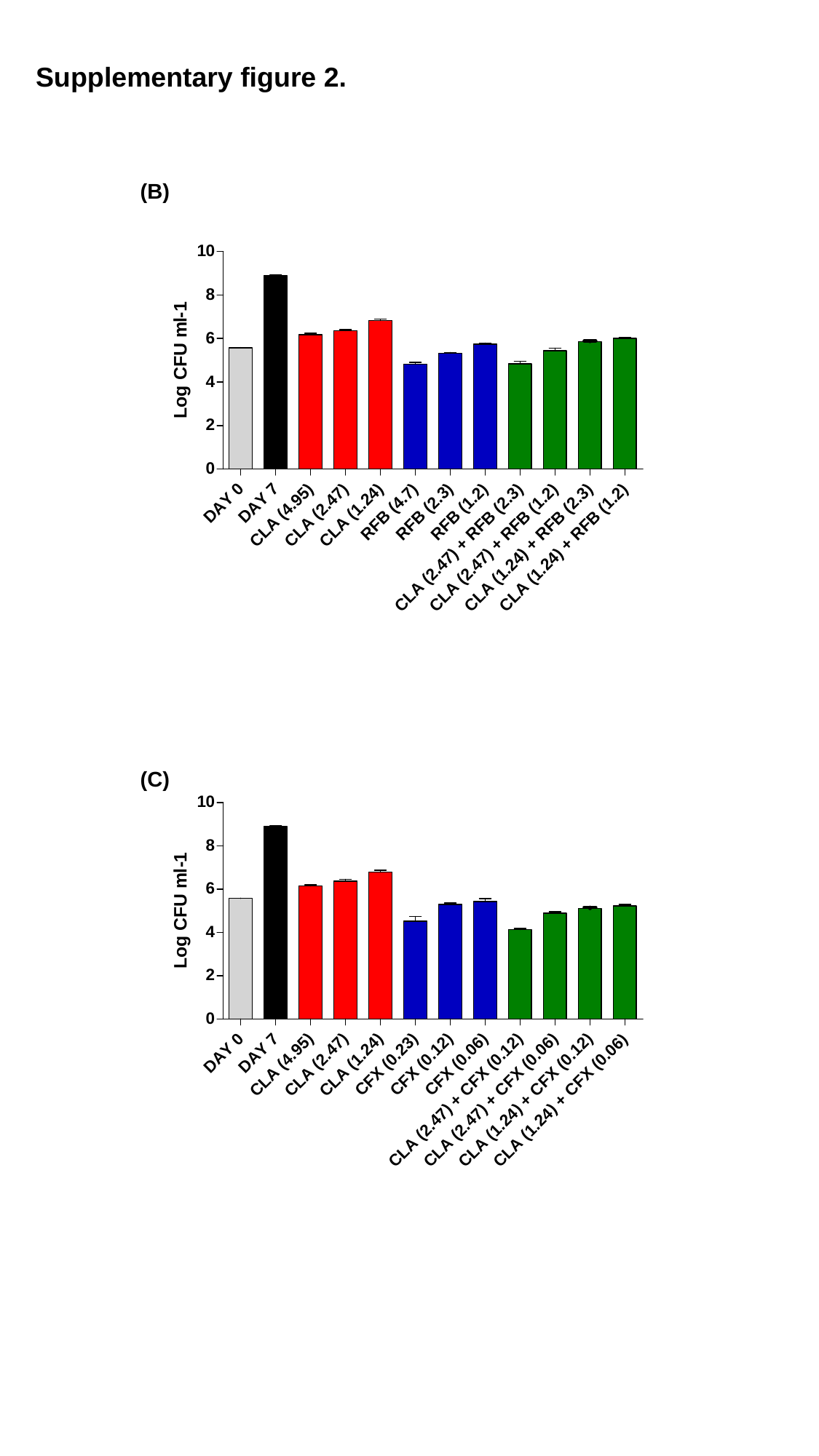

Supplementary figure 2.
(B)
(C)
